# Supplementary material for: WordCommentsAnalyzer: A windows software tool for qualitative research
Source: F1000Res. 2018 Sep 4;7:536. Originally published 2018 May 3. [Version 2] doi: 10.12688/f1000research.14819.2 (PMC6137411; doi:10.12688/f1000research.14819.2)
Supplement: Supplementary file 3 [file f1000research-7-17614-s0002.tgz › b2a79496-4ada-47c4-9682-a99ce867836d.zip!/Eight Iranian Health Journals/Journal of Knowledge and Health_32.docx]

© Journal of Knowledge and Health. This work is licensed under CC BY 4.0 (https://creativecommons.org/licenses/by/4.0/).

Volume & Issue: دوره 5، شماره 3-2: 1389

Title: ارزیابی میزان سمیت سه گیاه تاج خروس، بندواش و بابا آدم با استفاده از آزمون آرتمیا سالینا

http://knh.shmu.ac.ir/index.php/site/article/view/136

مقدمه: تاج خروس یا آشکنی به­صورت علف هرز در مراتع ایران می­روید. این گیاه توسط افراد بومی منطقه در غذاهای سنتی استفاده می­گردد. بندواش یا سگ­واش با سرعت رشد و انتشار بسیار بالا، آفتی خطرناک برای یونجه­زارها به شمار می­آید و موجب سندرم ترومورژنیک در گاو و گوسفند می­شود. «بابا آدم» که در بسیاری از مناطق ایران می­روید به­عنوان گیاه دارویی استفاده می­گردد. در اين مطالعه ميزان سميت سه گياه تاج خروس، بندواش و بابا آدم با استفاده از آزمون آرتمیا سالینا مورد بررسي قرار گرفت. مواد و روش­ها: این گیاهان از منطقه قائمشهر جمع­آوری و پس از خشک نمودن، عصاره این گیاهان تهیه شد، سپس سیست­های آرتمیا سالینا در آب درياي مصنوعي و در 26 درجه سانتي­گراد تفریج گردید و عصاره گیاهان در غلظت­های مختلف (ppm1000 و ppm 100 و ppm 10) با لاروهای آرتمیا مواجه شدند. میزان غلظتی که نیاز است تا نیمی از لاروها را از بین ببرد (LC50) با استفاده از معادله رگرسیون محاسبه گردید. نتایج: میزان  LC50سه گیاه تاج خروس، بندواش و بابا آدم به­ترتیب ppm 1640، ppm 990 و ppm 840 بود. نتیجه­گیری: براساس نتایج حاصله گياه بابا آدم نسبت به دو گونه ديگر داراي اولویت بيش­تري جهت مطالعه تكميلي و شناسايي تركيبات سمي می­باشد.

Title: بررسی عوامل مؤثر بر استعمال دخانیات براساس مدل بزنف در دانشجویان پسر مشغول به تحصیل شهر کرمانشاه

http://knh.shmu.ac.ir/index.php/site/article/view/137

مقدمه: به­دلیل پیشرفت سریع شیوع مصرف سیگار در نوجوانان و جوانان و نیاز به شناخت مشکل، جهت حساس کردن جامعه و دست­اندرکاران مسایل بهداشتی، این مطالعه با هدف تعیین عوامل مؤثر بر استعمال دخانیات براساس مدل بزنف در دانشجویان پسر شهر کرمانشاه انجام شد. مواد و روش­ها: در این مطالعه مقطعی تعداد 568 نفر از دانشجویان پسر مشغول به تحصیل شهر کرمانشاه در سال 1387به روش نمونه­گیری گلوله برفی انتخاب و داده­ها توسط پرسش­نامه پژوهشگر ساخته پایا و روا، مشتمل بر 70 سؤال در 5 جزء شامل سوالات دموگرافیک، آگاهی، نگرش، قادر کننده و نرم­های norms)) انتزاعی گردآوری گردید. داده­ها با استفاده از آزمون کای- دو و آنالیز واریانس یک­طرفه تجزیه و تحلیل شدند. نتایج: میانگین نمره آگاهی در این پژوهش در خصوص استعمال دخانیات 1/7±3/89 و میانگین نمره نگرش 2/6 ± 7/72 به­دست آمد. آزمون­های آماری ارتباط معناداری را بین تعداد نخ سیگار مصرفی با دانشگاه و مقطع تحصیلی نشان نداد. بیش­ترین فراوانی عوامل تحریک کننده استعمال دخانیات، دسترسی آسان به سیگار و مهم­ترین نرم انتزاعی مؤثر در کشیدن سیگار، دوستان سیگاری به­دست آمد. رایج­ترین باور در استعمال سیگار در دانشجویان گروه هدف، رفع عصبانیت (3/38%) و نداشتن تفریحات سالم، تفنن و کنجکاوی مهم­ترین علت مصرف سیگار در اولین تجربه استعمال دخانیات آنان بوده است. نتیجه­گیری: در این بررسی ارتباط بین داشتن دوستان سیگاری به­عنوان بارزترین عامل در گرایش به سیگار، نقش فشار در گروه­های همسان را بیش­تر مشخص می­سازد. دومین عامل مؤثر در دامنه نرم­های انتزاعی، والدین سیگاری بودند. در مرحله بعد اساتید و شخصیت­های تلویزیونی به عنوان عامل اثرگذار بر قصد دانشجویان در استعمال سیگار محسوب شدند.

Title: بررسی شاخص توده بدنی و فعالیت بدنی در دانش¬آموزان پسر 11 تا 14 ساله شهر خوشاب

http://knh.shmu.ac.ir/index.php/site/article/view/139

مقدمه: هدف از تحقیق حاضر بررسی شاخص توده بدنی و فعالیت بدنی در نوجوانان پسر 14-11ساله شهر خوشاب مي­باشد. مواد و روش­ها: اين مطالعه مقطعي روی 299 نوجوان پسر 14-11 ساله شهر خوشاب انجام شد. نمونه­هاي آماري از تمامي مدارس راهنمايي شهر به­صورت تصادفي انتخاب شدند. وضعیت لاغري، اضافه وزن و چاقی براساس شاخص توده بدنی برای سن و جنس در مقایسه با صدک­های استاندارد مركز كنترل بيماري­ها (CDC-2000) ارزیابی گردید. سطح فعالیت بدنی با استفاده از پرسش­نامه بین­المللی فعالیت بدنی ارزیابی شد. رفتارهای کم­تحرک آزمودنی­ها و سطح تحصیلات والدین آزمودنی­ها با استفاده از پرسش­نامه محقق ساخته ارزیابی شد. نتايج: شیوع لاغری و اضافه وزن- چاقی در بین آزمودنی­ها به­ترتیب 16% و 4/3% بود. میانگین زمان فعالیت بدنی سبک، متوسط و شدید در دانش­آموزان چاق و داراي اضافه وزن به­ترتیب 19، 24 و 15 دقیقه، در آزمودنی­های با وزن طبيعي به­ترتیب 19، 44 و 36 دقیقه و در آزمودنی­های لاغر به­ترتیب 22، 60 و 41 دقیقه در شبانه­روز بود. ميانگين سطح فعالیت بدنی در گروه اضافه وزن- چاق نسبت به گروه لاغر و گروه با وزن طبیعی، کم­تر ولی هیچ تفاوت معناداری بین آن­ها مشاهده نشد و میزان تماشای تلویزیون در گروه اضافه وزن- چاق نسبت به گروه با وزن طبیعی به­طور معناداری بیش­تر بود. سطح تحصيلات والدين در گروه اضافه وزن- چاق به­طور معناداري نسبت به گروه با وزن طبيعي بالاتر بود. نتیجه­گیری: یافته­های این مطالعه نشان داد که شیوع لاغری نسبت به شیوع اضافه وزن و چاقی در نوجوانان خوشابی بیش­تر می­باشد. در این مطالعه ارتباطی بین میزان فعالیت بدنی و شاخص توده بدنی مشاهده نشد، اما افزایش رفتارهای کم­تحرک (تماشای تلویزیون) با اضافه وزن و چاقی مرتبط بود.

Title: مقايسه ترکيب بدني دانشجويان پسر داراي فعاليت بدني منظم و غيرفعال دانشگاه صنعتي شاهرود

http://knh.shmu.ac.ir/index.php/site/article/view/140

مقدمه: تحقيقات، چاقي را منشأ بيماري­هايي از جمله سكته­ي قلبي، ديابت نوع دوم، فشار خون، برخي سرطان­ها، بيماري­هاي مفصلي و ريوي مي­دانند. در ميان عوامل ترکيب بدني درصد چربي، نمايه­ي توده بدن و نسبت دور کمر به دور باسن مهم­ترين عوامل اثرگذار بر تندرستي معرفي شده­اند. هدف اين تحقيق توصيف و مقايسه برخي عوامل ترکيب بدني دانشجويان پسر فعال و غيرفعال دانشگاه صنعتي شاهرود مي­باشد. مواد و روش­ها :در اين تحقيق70 دانشجوي غيرفعال (دانشجوياني که برنامه ورزشي و فعاليت جسماني منظمي نداشته­اند) با ميانگين سني 7/1±9/20 سال و 65 دانشجوي داراي فعاليت بدني منظم با ميانگين سني 6/1±8/20 سال به­صورت تصادفي طبقه­اي براساس دانشکده و سال ورود از بين 2013 دانشجوي مشغول به تحصيل در سال 1386 انتخاب و شاخص­هاي ترکيب بدني آن­ها به­وسيله دستگاه آناليز ترکيب بدني به روش آناليز امپدانس الکتريکي تعيين گرديد. نتايج: نتايج نشان داد در دانشجويان فعال ميانگين درصد چربي بدن 2/3±4/13 و دانشجويان غيرفعال 2/6±7/16 بود که اختلاف آن­ها از نظر آماری معنادار (001/0P<) بود. در مقايسه توده بدون چربي: دانشجويان فعال 1/8 ±3/60 کيلوگرم و دانشجويان غيرفعال 6/7±4/56 کيلوگرم بود که اختلاف بين دو گروه معنادار (005/0(P= بود در نمايه­ي توده بدن دانشجويان فعال داراي ميانگين 8/2±6/22 و دانشجويان غيرفعال 8/3±3/22 بودند ولي تفاوت معناداري (519/0P=) بين دو گروه وجود نداشت. در شاخص نسبت دور کمر به دور باسن دانشجويان فعال داراي ميانگين 03/0±8/0و دانشجويان غيرفعال 06/0±81/0بودند که بين دو گروه تفاوت معناداري وجود نداشت. نتيجه­گيري: براساس يافته­هاي اين تحقيق مي­توان نتيجه گرفت که تغييرات درصد چربي و وزن خالص که از عوامل مؤثر بر تندرستي هستند، مرتبط با فعاليت جسماني مي­باشند.

Title: تأثير روش‌هاي مختلف آموزش فعاليت بدني در كودكان و نوجوانان مبتلا به اضافه وزن

http://knh.shmu.ac.ir/index.php/site/article/view/142

مقدمه: با توجه به روند روز افزون چاقي و تأثير كاهش فعاليت بدني در افزايش بروز اضافه وزن كودكان و نوجوانان و محدوديت خانواده­ها براي رفت و آمد و شرکت فرزندان در كلاس­هاي ورزش، دست­يابي به شيوه مناسب ارايه آموزش فعاليت بدني در منزل ضروري به­نظر مي­رسد. مواد و روش­ها: اين مطالعه مداخله­اي غير دارويي، به مدت شش ماه از ارديبهشت تا آبان 1386 در بين 105 نفر از كودكان و نوجوانان 18-6 ساله مبتلا به چاقي كه به­طور تصادفي در سه گروه 35 نفري قرار گرفتند انجام شد. تغذيه و رفتار درماني در همه گروه­ها به­صورت يكسان ارايه گردید ولي آموزش فعاليت فيزيكي در گروه اول به­صورت شركت در كلاس­هاي آموزش فعاليت فيزيكي 2 بار در هفته، در گروه دوم با در اختيار قرار دادن CD آموزشي و در گروه سوم آموزش به­صورت چهره به چهره انجام شد. قبل و بعد از مداخله شاخص­هاي آنتروپومتريك اندازه­گيري و ثبت شد. نتايج: ميانگين نمايه توده بدني (BMI) در افراد شركت­كننده در كلاس­هاي فعاليت فيزيكي و گروه تحت آموزش با CD، بعد از مداخله به­طور معناداري نسبت به قبل از مداخله كاهش يافته بود ولی اختلاف معناداری بین سه روش مختلف آموزش فعالیت بدنی مشاهده نشد. نتيجه­گيري: يافته­ها نشان داد روش آموزش با CD نيز در كاهش BMI كودكان و نوجوانان مبتلا به اضافه وزن و چاقي به اندازه شيوه آموزش چهره به چهره و شركت در كلاس فعاليت بدني، تأثيرگذار است. تعميم اين قبيل مداخلات مي­تواند در سطح جامعه مؤثر باشد.

Title: محاسبه هزينه تمام شده خدمات ارایه شده در مراكز بهداشتي- درماني و خانه هاي بهداشت شهرستان شاهرود

http://knh.shmu.ac.ir/index.php/site/article/view/144

مقدمه: محاسبه هزينه­ها ابزار مديريتي مهمي جهت برنامه­ريزي، كنترل، نظارت و ارزشيابي خدمات سلامت است كه به­منظور تصميم­گيري آگاهانه انجام مي­شود. اين مطالعه به­منظور تعیین هزينه تمام­شده خدمات ارایه شده در مراكز بهداشتي- درماني و خانه­هاي بهداشت شهرستان شاهرود در سال 1388 انجام شد. مواد و روش­ها: در اين مطالعه كاربردي- توصيفي کلیه مراکز بهداشتی -درمانی شهری و روستایی به روش سرشماری انتخاب و اطلاعات مورد نياز در قالب 70 فرم جمع­آوري داده­ها جهت خدمات، مقدار مواد مصرفي اختصاصي و عمومی هر خدمت، دارو و تجهيزات، زمانبري هر خدمت و فعاليت، هزینه استهلاک ساختمان­ها و تجهیزات، گردآوري و سپس هزينه­هاي هر واحد خدمت مشتمل بر هزينه­هاي مستقيم و غيرمستقيم (سرباري)، هم­چنین هزینه یک مرکز و خانه بهداشت، با استفاده از نرم­افزار تحلیل هزینه محاسبه گرديد. نتايج: یافته­های حاصل از تحلیل داده­ها نشان داد که 4/44% ارایه­دهندگان را مردان و 6/55% را زنان تشکیل می­دادند. 8/22% از نیروها در خانه­های بهداشت، 1/26% در مراکز بهداشتی- درمانی روستایی، 1/9% در مراکز بهداشتی- درمانی شهری، 5/24% در مراکز بهداشتی- درمانی شبانه­روزی شهری، 6/2% در پایگاه­های بهداشتی و 9/14% در ستاد معاونت بهداشتی شهرستان شاغل بودند. بيش­تر هزينه­ها مربوط به هزینه­های پرسنلی با 1/66% و پس از آن هزینه ستادی شهرستان با 8/12%، دارو با 0/11% و مصرفی اختصاصی با 8/3% قرار داشتند. بالاترین هزینه خدمت مربوط به آموزش بهورز با 1325209 ريال و کم­ترین آن مربوط به نمونه­گیری آنفلوانزا با 3872 ریال بود. نتيجه­گيري: با توجه به بالابودن هزینه­هاي پرسنلي، افزايش بهره­وري نيروي كار نقش مهمي در كاهش مخارج خواهد داشت. هم­چنين تعدیل نیروها و استفاده از مشارکت بخش خصوصی در ارایه خدمات و برون­سپاری واحدهاي پرهزينه، می­تواند در استفاده بهینه از منابع نقش مهمی را ایفا نماید

Title: بررسی ناراحتی¬های اسکلتی- عضلانی و عوامل مرتبط در کارگران یکی از میدان¬های نفتی جنوب

http://knh.shmu.ac.ir/index.php/site/article/view/146

مقدمه: ناراحتی­های اسکلتی- عضلانی یک مشکل روبه رشد در جهان است كه به­دلیل ناتوانی­های طولانی مدت، هزینه­های زیادی را بر دوش سازمان­ها و دولت­ها می­گذارد. کارکنان میدان­های نفتی که نقش مهمی در اقتصاد کشور دارند به­دلیل وضعیت­های استاتیک، تکراری و استرس­زا در کار با ماشین آلات حفاری، بر روی دکل­ها و سکوها اغلب با خطر ناراحتی­های اسکلتی- عضلانی مواجه هستند. هدف این مطالعه، بررسی ناراحتی­های اسکلتی- عضلانی و عوامل مرتبط در این کارگران می­باشد. مواد و روش­ها: نوع مطالعه توصیفی- تحلیلی بوده و جامعه مورد مطالعه 60 نفرکارگران یکی از میدان­های نفتی جنوب در سال 1386 می­باشند که در بخش­های حفاری (سکوبان، دکلبان و .....)، تعمیرات و نگهداری (جوشکار، برقکار و ...)، خدمات (نظافتچی، آشپز و ...) و مهندسی (نمودارگیر گل حفاری و ...) کار می­کنند. داده­ها با پرسش­نامه استاندارد نورديك به روش مصاحبه جمع­آوري و تجزیه و تحلیل اطلاعات با استفاده از آزمون­های χ2 و رگرسیون لجستیک انجام شد. نتایج: در این مطالعه کارگران با ميانگين سني 8/9± 0/ 35 شرکت کردند و 3/48% از آنان بیش از 5 سال سابقه کار داشتند. از عوامل فیزیکی کار 3/83% از کارکنان در شغل خود حرکات تکراری مچ و انگشتان، خم شدن تکراری آرنج و کارکردن با دست در ارتفاع بالاتر از شانه و 3/88% بلند کردن بار بیش از 25 کیلوگرم را گزارش کردند. در این مطالعه 7/86% حداقل بک ناراحتی اسکلتی- عضلانی را در 12 ماه گذشته گزارش کردند. شایع­ترین ناراحتی زانودرد (1/71%) و سپس کمردرد (7/61%) و شانه­درد (7/51%) به­دست آمد. تحلیل رگرسیون نشان داد بین کمردرد با حمل بار، درد شانه و آرنج با تحصیلات و زانودرد با شغل حفاري ارتباط معناداری وجود دارد (05/0P<). نتیجه­گیری: با توجه به نتایج، ناراحتی­های اسکلتی عضلانی در کارگران این میدان نفتی بالاست. برای پیشگیری اقدامات فوری مداخله­ای ارگونومیک پیشنهاد می­گردد.

Title: بررسی عوامل خطر ابتلا به آنفلوانزای پاندمیک H1N1

http://knh.shmu.ac.ir/index.php/site/article/view/147

مقدمه: آنفلوانزاي پاندميک H1N1 يک بيماري عفوني حاد تنفسي است که باز ترکيبي از دو نوع ويروس آنفلوانزاي نوع A است. این مطالعه با هدف شناسایی عوامل خطر ابتلا به این بیماری نوپدید انجام شده است. مواد و روش­ها: در اين مطالعه مورد شاهدی، 18 مورد مثبت آنفلوانزاي پاندميك H1N1 به­عنوان مورد و 72 بيمار بخش­هاي ارتوپدي، ارولوژي، جراحي و زنان در فاصله زماني 15 مرداد تا 30 آذر 88 به­طور تصادفي به­عنوان شاهد انتخاب شدند. داده­ها از طریق یک فرم و توسط دو نفر پرستار با تجربه جمع­آوری شدند. داده­ها وارد نرم­افزار SPSS شده و با استفاده از آزمون­های t مستقل و کای اسکوئر مورد تحلیل قرار گرفتند. نتایج: بین ابتلا به آنفلوانزای پاندمیک H1N1 و سابقه سفر داخلی، تماس با بیمار مبتلای آنفلوانزایی، بیماری­های تنفسی، دیابت، ارتباط معنادار نشان داده شد (05/0P<) و با سابقه سفر خارجی، عفونت ثانویه، بیماری قلبی، پرفشاری خون، بیماری قلبی، بیماری عصبی، سابقه واکسیناسیون رابطه معناداری مشاهده نشد (05/0P>).نتیجه­گیری: افراد دارای بیماری­های زمینه­ای به­خصوص بیماری­های تنفسی، دیابت، بیماری قلبی و دارای عفونت­های ثانویه با احتمال بسیار زیادی در مقابل ابتلا و مرگ ناشی از آنفلوانزای پاندمیک H1N1 حساسیت دارند

Title: جداسازی انگل لیشمانیا تورانیکا از فلبوتوموس پاپاتاسی به¬وسیله تعیین توالی ناحیه (Internal Transcribed Spacer 1) ITS1 در کانون لیشمانیوز جلدی روستایی شهرستان دامغان، استان سمنان

http://knh.shmu.ac.ir/index.php/site/article/view/150

مقدمه: انگل لیشمانیا تورانیکا نیز از فلبوتوموس پاپاتاسی و جوندگان جدا شده که بیماری­زایی آن در انسان مشخص نیست. مواد و روش­ها: این مطالعه به­منظور تعیین آلودگی پشه خاکی­ها به انگل لیشمانیا تورانیکا در مناطق روستایی شهرستان دامغان انجام شده است. در اين مطالعه پشه خاکی­ها به­وسیله تله­های چسبان صید شده­اند. پشه خاکی­های ماده پس از شناسایی در فرایند استخراج DNA و جستجو برای انگل لیشمانیا در تکنیک Conventional PCR با استفاده از پرایمرهای ITS1 (LITSR و L5.8S) قرار گرفتند. محصولات واکنش تعیین توالی توسط نرم­افزارBlast  تجزيه و تحليل شدند. نتايج: 218 پشه خاکی فلبوتوموس پاپاتاسی مورد آزمایش واکنش زنجیره­ای پلیمرازی قرار گرفتند. یک مورد از انگل­های جداشده مربوط به گونه لیشمانیا تورانیکا بوده است (5/0%). این یافته نخستین بار در استان سمنان و دومین مرتبه در ایران ثبت شده است. نتيجه­گيري: بیماری­زایی لیشمانیا تورانیکا در انسان مشخص نیست اما در موش­های آزمایشگاهی باعث بیماری می­شود. مطالعات نشان می­دهند در آلودگی همزمان جوندگان به انگل­های لیشمانیا تورانیکا و ماژور، قدرت بیماری­زایی لیشمانیا ماژور افزایش می­یابد. این نشان می­دهد شاید ایمنی برخی افراد به لیشمانیوز جلدی در مناطق آندمیک به­علت مواجهه با لیشمانیا تورانیکا باشد و این گونه می­تواند به­عنوان کاندیدی جهت ساخت واکسن مدنظر قرار گیرد.
